# A detergent-based procedure for the preparation of IgG-like bispecific antibodies in high yield

**DOI:** 10.1038/srep39198

**Published:** 2016-12-16

**Authors:** Jyoti Gupta, Mehboob Hoque, Masihuz Zaman, Rizwan Hasan Khan, M. Saleemuddin

**Affiliations:** 1 Interdisciplinary Biotechnology Unit, Aligarh Muslim University, Aligarh 202002, India

## Abstract

Bispecific antibodies (BsAbs), with the ability to recognize two different epitopes simultaneously, offer remarkable advantages in bioassays, cancer therapy, biosensors, and enzyme electrodes. Preparation and purification of BsAbs in adequate quantities remains a major hurdle in their use in various applications. Poor yield is also the principal limitation in the preparation of BsAbs by the redox procedure. IgG with reduced inter-heavy chain disulfides do not dissociate into half molecules at neutral pH. In this study, we report that the dissociation occurs in presence of sodium dodecyl sulphate (SDS) and inclusion of the detergent during the redox procedure results in remarkable increase in the formation of the BsAbs. Exposure of antibodies to 0.1% (w/v) SDS causes only minor loss in secondary/tertiary structure and the ability to bind the antigen. The BsAbs prepared using the modified redox procedure that recognize the antigens HRP and α-LA were prepared and successfully employed for detecting α-LA in milk/dairy products by ELISA and dot blot techniques. BsAbs were also prepared from partially purified immunoglobulin gamma (IgG). This work shows for the first time that SDS, by dissociating IgG with reduced inter-heavy chain disulfides into half molecules, markedly enhances the formation of BsAbs by the redox procedure.

Therapeutic potential of antibodies is now widely recognized and several monoclonal antibodies have markedly advanced the treatment of some cancers as well as other human diseases^1^. Among the attempts made to increase the clinical efficacy of antibodies, conversion to bispecific antibodies (BsAbs) is prominent^2^. BsAbs recognize/bind two different epitopes on the same or different antigens, have the potential to direct immune effector cells such as natural killer cells and T-cells to tumor cells and thereby facilitate the destruction of the later^3^. BsAbs also have immense potential in clinical diagnosis^4^,^5^. In spite of the recognition of the remarkable potential of BsAbs, difficulties in their production and purification in adequate quantities continues to remain a challenge. Two BsAbs, catumaxomab (Removab^®^, anti-EpCAM × anti-CD3) and blinatumomab (Blincyto^®^, anti-CD19 × anti-CD3) have been approved for therapy^6^ and more than twenty BsAbs have entered clinical trials^7^.

BsAbs can be prepared by chemical conjugation of two antibodies (or fragments derived thereof), fusion of two antibody producing cell lines or genetic approaches resulting in the recombinant molecules. While chemical conjugation was the first and simplest strategy to generate the bispecifics^8^, hybrid-hybridoma technology is currently most widely used^9^. Time consuming tissue culture methodology, high heterogeneity and low yield of the produced BsAbs as well as the need for multiple affinity purifications add to the cost of the finished product. The genetic approach on the other hand suffers from the need for expensive experimental set up and poor product yields.

Two major classes of BsAbs are currently under investigation: the IgG like BsAbs (that have structures similar to the IgG) and small BsAbs that lack the fragment crystallisable region (Fc)^10^. The Fc region facilitates affinity purification of BsAbs (on Protein A or protein G columns), helps in improving their stability, enhances circulating half-life, antibody dependent cell mediated cytotoxicity (ADCC) and complement fixation (CDC).

There have been several attempts to chemically join half antibody molecules, fragment antigen-binding (Fab’) fragments and even intact antibodies through inter chain disulfide linkages or using bifunctional crosslinkers to generate BsAbs^11^,^12^,^13^,^14^. The procedures give far lower than the theoretical yields, principally due to poor specificity of the crosslinking reactions. Chemical conjugation procedures are more efficient in producing bispecifics formed from antigen binding fragments like the Fab’s, rather than in the generation of IgG like BsAbs, because of the presence of strong interactions between the Fc regions of the two heavy chains that interfere with the dissociation of the two half molecules and consequently in the formation of the bifunctionals^15^. More recently a redox procedure has been described by Carlring *et al*.^16^ in which a mixture of two different antibody molecules is subjected to reduction under the conditions causing the selective disruption of inter-heavy chain thiols followed by reoxidation of the thiols, to generate BsAbs in moderate yield. This study reports a high yield procedure for the preparation of BsAbs, suitable for the preparation of the bifunctionals with IgG format also from partially purified antibody preparations.

## Results

### Generation of antibody half molecules

**Figure 1:**
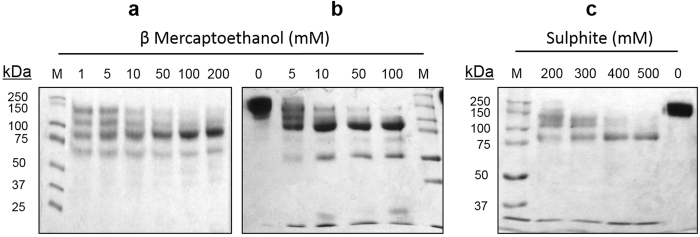
Reduction of rabbit and goat IgG with thiol reductants. Rabbit IgG (Panel a) and goat IgG (Panel b) were treated with various concentrations of β-ME for 1 h at 37 °C and subjected to SDS-PAGE under non-reducing condition. Rabbit IgG was treated with various concentration of sodium sulphite and 2.5 mM DTNB for 2 h at 37 °C, dialysed and subjected to SDS-PAGE under non-reducing condition (Panel c). M, marker proteins. Full length gel images are presented in Supplementary Fig. S1.

Anti-α-lactalbumin (α-LA) and anti-horseradish peroxidise (HRP) antibodies raised in rabbits were used as model antibodies, the later also for cutting down the BsAb assay time during ELISA^17^. The inter-heavy chain disulfide bonds in the rabbit IgG are more susceptible to reduction than those between heavy and light chain^18^. Conditions for the selective reduction of inter-heavy chain disulfides were therefore worked out. When exposed to 10 mM β-mercaptoethanol (β-ME), rabbit IgG migrate in SDS-PAGE mainly as a 75 kDa peptide, suggesting the formation of half molecules (Fig. 1a). Small amounts of peptides corresponding to 50 and 25 kDa were also evident. As shown in Fig. 1b goat IgG also give rise to half molecule-like preparation at the same concentration of the reductant. At lower β-ME concentrations (≤5 mM), multiple bands, suggesting the formation of peptides containing heavy-heavy/heavy-heavy-light chains, were noticeable (Fig. 1a lane 2 & 3 & 1b lane 2). When exposed to 500 mM sulphite and 2.5 mM 5,5-Dithiobis (2-nitrobenzoic acid) (DTNB), rabbit IgG also migrate in SDS-PAGE mainly as a 75 kDa peptide.

### Gel filtration behaviour of IgG with reduced inter-heavy chain disulfides

**Figure 2:**
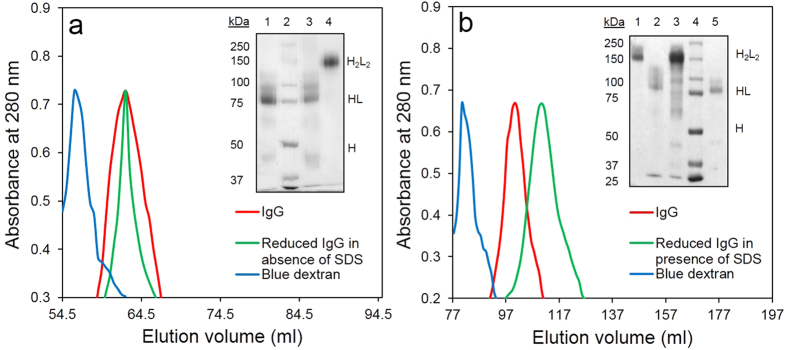
Gel filtration chromatography of rabbit IgG and the inter-heavy chain-reduced IgG in presence and absence of 0.1% (w/v) SDS. Panel a: A 150 ml Sephacryl S-200 column equilibrated with Tris buffered saline (25 mM Tris, 1 mM EDTA, 0.1 M NaCl, pH 8.8) was used. Native rabbit IgG and those reduced with sodium sulphite in presence of DTNB were passed through the column. Inset shows non-reducing SDS-PAGE analysis. Lane 1 and 4, eluted peak fractions collected after loading reduced IgG and native IgG respectively, lane 2 molecular weight marker and lane 3 reduced IgG not subjected to gel filtration. Panel b: A 250 ml Sephacryl S-300 column equilibrated with Tris buffered saline, pH 8.8, equilibrated with 0.1% SDS was used and samples loaded as described for panel a. Inset shows the non-reducing SDS-PAGE analysis. Lane 1 native IgG, lane 2 reduced IgG not subjected to gel filtration, lane 3 native IgG eluted from the column, lane 4 molecular weight markers and lane 5 peak fraction collected after loading reduced IgG. Full length gel images are presented in Supplementary Fig. S1.

It is well recognised that antibody heavy chains are associated, in addition to the disulfides, with strong non-covalent interactions in the Fc region^19^. Reduction of the inter-heavy chain disulfide linkages of IgG derived from humans^20^ and rabbits^21^ may therefore not result in their dissociation into half molecules. To further examine the effect of reduction of inter-heavy chain disulfide on the dissociation of antibody molecules into two halves, rabbit IgG treated with 500 mM sodium sulphite and DTNB were subjected to gel filtration chromatography both in absence or presence of 0.1% (w/v) SDS. The free thiol groups in the reduced antibody molecules were blocked with DTNB in order to prevent their reoxidation during the chromatography. As shown in Fig. 2a, in absence of SDS, the reduced and DTNB-protected rabbit IgG eluted from the Sephacryl S-200 column with an elution volume identical with the IgG not exposed to the thiol reductant, suggesting lack of dissociation of the reduced IgG into half molecules. SDS-PAGE of the reduced IgG however yielded a major 75 kDa band (Fig. 2a inset). Gel filtration chromatography performed in presence of SDS however revealed that the reduced IgG emerged from the Sephacryl S-300 column with higher elution volume than the untreated IgG (Fig. 2b). The molecular weight of the IgG half molecules calculated from the gel filtration calibration curve in presence of SDS was 79 kDa (Supplementary Fig. S2), while it was 150 kDa in absence of the detergent (Supplementary Fig. S2). These experiments substantiate earlier studies which suggest that IgG molecules with reduced inter-heavy chain disulfide do not readily dissociate into half molecules. The strong interactions between the CH3 domains in the Fc region of IgG is now well recognized^22^. The natural occurrence of IgG4 as functional monospecific antibodies for a given antigen is attributed to weak non-covalent interaction between the CH3 domains of heavy chains that facilitates half antibody exchange^23^ and increased susceptibility of the hinge disulfide bonds to reduction.

### Hydrodynamic radii of native IgG and half molecules

**Figure 3:**
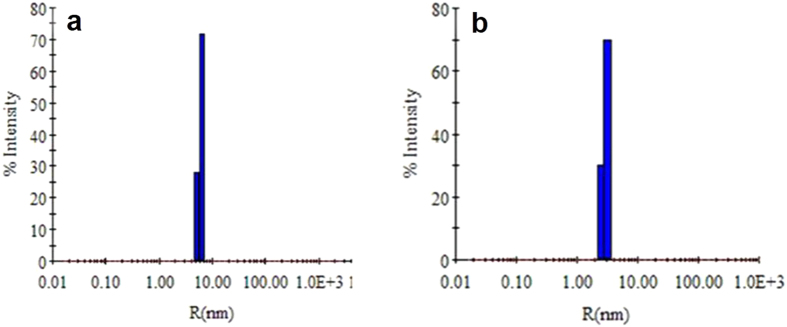
DLS pattern for native IgG (Panel a) and half molecules in presence of 0.1% (w/v) SDS (Panel b). Values expressed are the best representative of three independent experiments.

In order to determine the size and homogeneity of IgG and half molecules, their dynamic light scattering (DLS) in solution was recorded. Figure 3a,b shows the hydrodynamic radius (R_h_) value obtained for native IgG and DTNB treated half molecules in presence of SDS. As summarized in the table (Supplementary Table S1) native IgG showed R_h_ of 5.4 nm and that for half molecules was 3.4 nm. The molecular weight obtained corresponding to R_h_ value indicate native IgG to be of 151 kDa and half molecules 79 kDa. When R_h_ is plotted against percent light intensity scattered, a distribution for particles size is obtained and the peak width at half its height corresponds to polydispersity. Polydispersity is therefore a representative of protein particle size distribution width. Percent polydispersity is polydispersity/R_h_ and values of percent polydispersity <20 indicate monodispersity and uniformity of size of protein particles in solution^24^. Both for IgG and half molecules, the percent polydispersity was <20, which indicates that the preparations were homogenous in terms of protein particle size. These results corroborate our results obtained from SDS-PAGE (Fig. 1) and gel filtration (Fig. 2).

### IgG are resilient to 0.1% (w/v) SDS exposure

**Figure 4:**
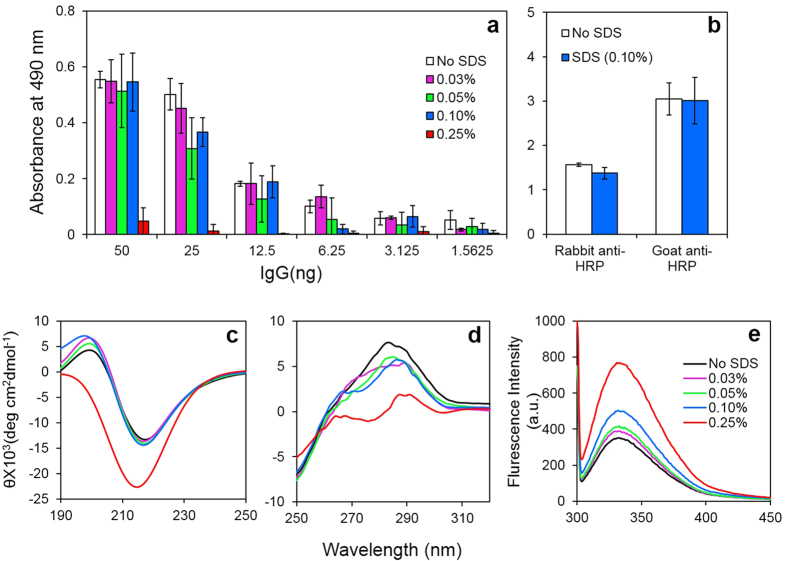
Effect of exposure to SDS on structure and function of IgG. The IgG fraction purified from the sera of rabbit immunized with α-LA was exposed to various concentrations of SDS, dialyzed and antigen binding activity assayed by ELISA (Panel a). Panel b shows the effect of 0.1% SDS on the antigen binding activity of anti-HRP IgG raised in rabbits and goats. Results are expressed as mean ± SD of three independent experiments. Rabbit IgG were incubated with various concentrations of SDS (detailed in Panel e also for Panel c,d) for 1 h, dialysed extensively against PBS, pH 7.4 and subjected to far-UV CD (Panel c), near-UV CD (Panel d) and intrinsic fluorescence (Panel e) spectral analysis. Results are expressed as mean of three independent experiments.

It is well known that SDS disrupts the non-covalent interactions, both between heavy-heavy and heavy-light chains of the IgG molecules. Before proceeding with the experiments to prepare BsAbs in presence of SDS, studies were carried out to ascertain the resilience of the antibodies against exposure to the detergent. Figure 4a shows the effect of exposure of rabbit anti-α-LA to various concentrations of SDS and subsequent removal of the detergents by dialysis. SDS concentrations up to 0.1% were well tolerated, as evident from only minor loss of antigen binding activity; higher concentrations of the detergent were however detrimental. Rabbit anti-α-LA IgG exposed to 0.1% SDS exhibited 98% antibody activity as compared to the native IgG, while 0.25% SDS-treated IgG showed retention of only 10% activity (Fig. 4a). Similarly, rabbit anti-HRP and goat anti-HRP IgG exposed to 0.1% SDS retained 90% and 98% activities, respectively when compared to respective native antibodies (Fig. 4b). Spectroscopic studies also support structural stability of the antibodies against SDS-exposure. Rabbit IgG exposed to 0.1% SDS exhibited only minor alterations in secondary and tertiary structure as evident from far, near-UV CD and intrinsic fluorescence spectroscopy (Fig. 4c–e). Goat IgG were far more resilient to transient exposure to 0.1% SDS as revealed by less marked alteration in far, near-UV CD and intrinsic fluorescence spectra (Supplementary Fig. S3).

### BsAb formation by the redox procedure is enhanced by inclusion of SDS

**Figure 5:**
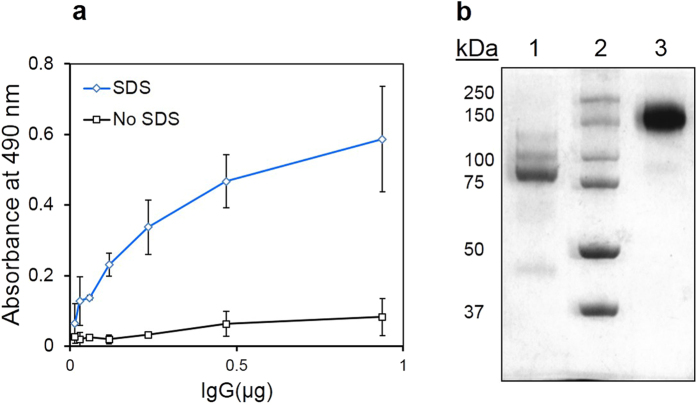
Preparation of BsAbs using anti-α-LA and anti-HRP antibodies, affinity purified from the sera of immunized rabbits. The immuno affinity purified anti-α-LA and anti-HRP antibodies were reduced separately with 10 mM β-ME for 1 h and mixed in 1:1 ratio in presence of 0.1% (w/v) SDS or absence of the detergent and dialysed against PBS, pH 7.4. Formation of the BsAbs was determined by ELISA (Panel a). Results are expressed as mean ± SD of three independent experiments. The preparations thus obtained were subjected to non-reducing SDS-PAGE [Panel b, IgG reduced to half molecules (lane 1), molecular weight markers (lane 2) and fraction after dialysis (lane 3)]. Full length gel images are presented in Supplementary Fig. S1.

Anti-α-LA and anti-HRP antibodies were immunoaffinity purified as described in methods, and subjected to the redox procedure in absence or presence of SDS for the preparation of the BsAbs (containing one arm each recognizing α-LA and HRP). While both anti-α-LA IgG and the BsAbs bind to the ELISA plates coated with α-LA, plates containing only the later can bind to HRP and be therefore detected using the o-phenylenediamine dihydrochloride (OPD) assay. As evident from the Fig. 5a very small quantities of BsAbs were formed when the redox procedure was carried out in absence of SDS. Inclusion of the detergent however resulted in a remarkable (over 6-fold) increase in the formation of the BsAbs. Figure 5b shows that IgG subjected to the redox procedure migrate in the SDS-PAGE as a single 150 kDa band suggesting complete reassociation of the half molecules.

### BsAbs can be prepared using partially purified antibodies by the modified redox procedure

**Figure 6:**
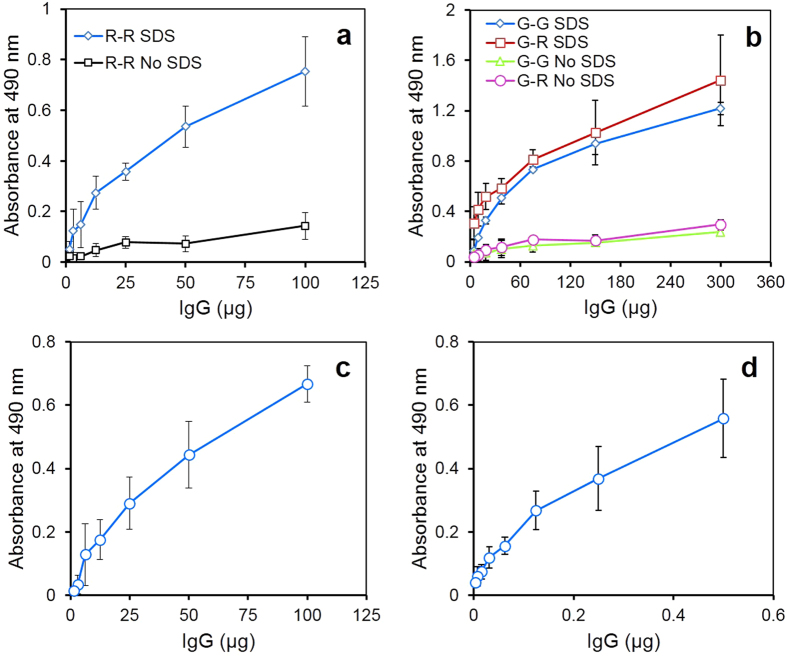
Preparation of BsAbs using the IEC fractions isolated from sera of rabbits and commercial goat IEC fraction as well as purification of rabbit anti-α-LA: anti-HRP BsAbs by twin affinity chromatography. Panel a: Rabbit anti-α-LA: rabbit anti-HRP BsAbs were prepared essentially as described from the immuno affinity fractions (legend to Fig. 5), and assayed for the presence of BsAbs by ELISA. Panel b: BsAbs recognizing HRP and HSA were prepared using goat anti-HSA and anti-HRP antibodies, in absence (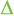) or presence of SDS (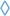). BsAbs were also prepared from goat anti-HSA and rabbit anti-HRP in absence (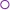) or presence of SDS (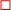) and assayed by ELISA. Activity of the BsAb prepared from the IEC fractions of rabbit anti-α-LA and anti-HRP antibodies before (Panel c) and after twin affinity purification (Panel d) in which fifty milligrams each of rabbit anti-α-LA and anti-HRP IEC fractions were subjected to reduction and oxidation in presence of 0.1% (w/v) SDS to obtain BsAbs and activity assayed by ELISA. Data are presented as the mean ± SD of three independent experiments.

BsAb formation during the redox procedures is expected to go down with increase in the heterogeneity of the antibody preparations used. For this reason, monoclonal/affinity purified polyclonal antibodies are generally used as starting material for preparing BsAbs. Encouraged by the remarkable increase in the yield of BsAbs prepared in presence of SDS, we attempted the preparation of BsAbs using the ion exchange chromatography (IEC) fractions of anti-sera raised in rabbits. As evident from the Fig. 6, the activity of BsAbs prepared using IEC fractions of anti-α-LA and anti-HRP antibodies by reduction and oxidation in absence of SDS was very low but increased over 8 times when the detergent was included during the preparation (Fig. 6a). BsAbs were also successfully prepared using ion exchange purified goat anti-HSA and goat anti-HRP antibodies (Fig. 6b). The procedure was also effective in the preparation of hybrid BsAbs using goat anti-HSA and rabbit anti-HRP antibodies. Incorporation of SDS resulted in 5 and 6 fold higher BsAb activity in case of goat: rabbit and goat: goat preparations respectively (Fig. 6b).

### Affinity Purification of BsAbs prepared from ion exchanged fractions

**Figure 7:**
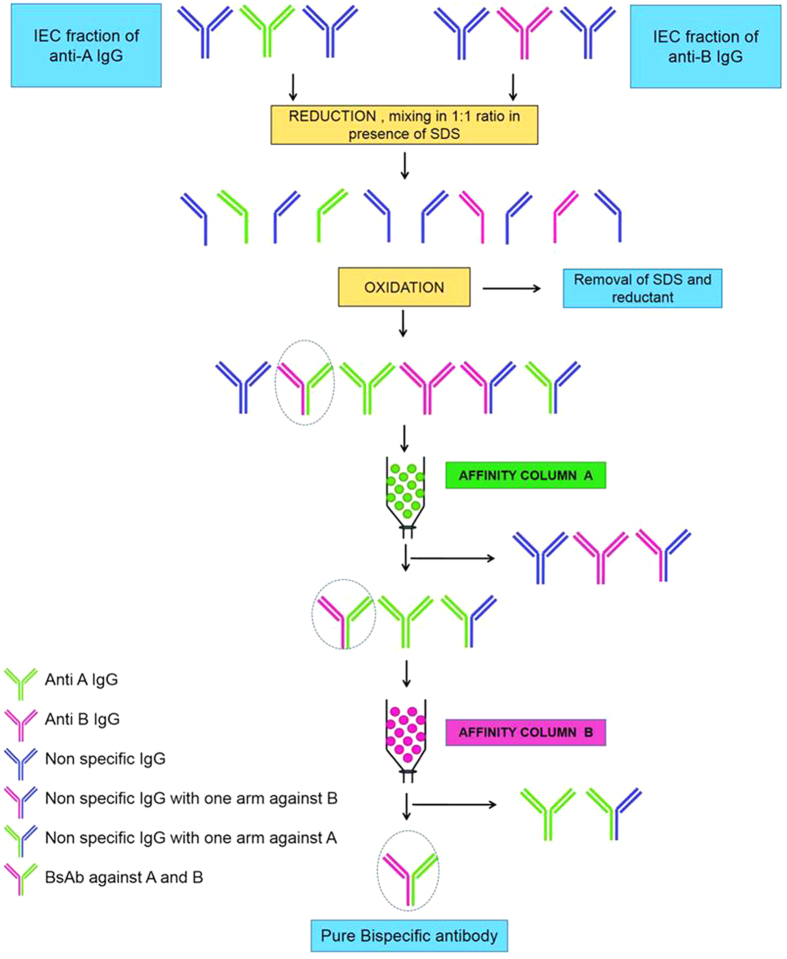
Schematic representation of the preparation and purification of BsAbs by the detergent-based redox procedure.

Samples containing mixtures of IEC purified anti-α-LA and anti-HRP IgG (50 mg each) carried through the redox procedure in presence of SDS, were passed successively through affinity columns of α-LA-Sepharose and HRP-Sepharose to purify the BsAbs (Supplementary Fig. S4). Only 1.16 mg of protein was eluted when the preparation was passed through α-LA-Sepharose column and the eluate when subsequently purified on HRP-Sepharose yielded 0.5 mg protein suggesting a protein yield of 0.5%. A comparison of the activities of the BsAbs before and after affinity purification suggested about 200-fold enrichment in antigen binding activity (Fig. 6d). A schematic presentation for purification of BsAbs from IEC fraction is illustrated in Fig. 7.

### Detection of α-LA in test samples using the BsAbs

**Figure 8:**
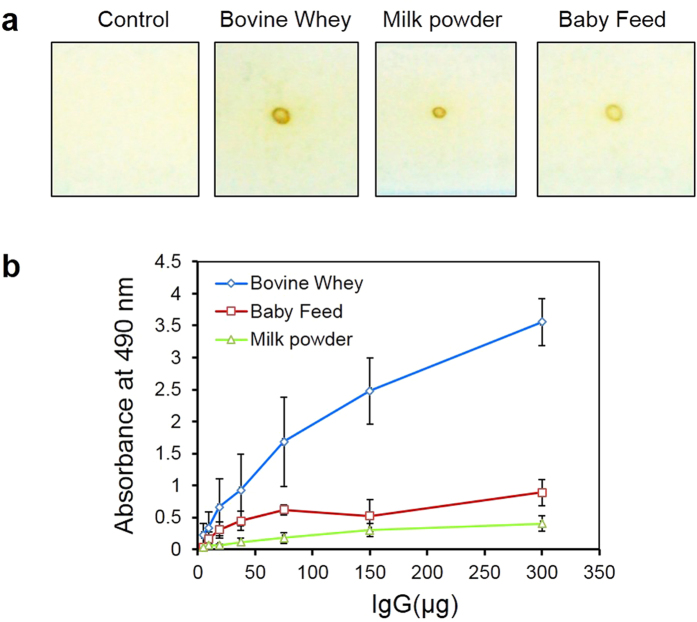
Assay of α-LA in whey, commercial milk powder and baby feed samples using rabbit anti-α-LA: anti-HRP BsAb prepared using the detergent-based redox procedure. Panel a: Samples containing 500 ng proteins were applied as dots, blocked with BSA and visualized using BsAb. Panel b: ELISA plates were coated with test samples containing 200 μg protein, blocked with BSA and visualized using the BsAb. Data are presented as the mean ± SD of three independent experiments.

α-LA in some dairy based food samples was detected by the in house prepared rabbit anti-α-LA: anti-HRP BsAbs, both by dot blot assay and ELISA. Test samples were coated onto the wells of ELISA plates or nitrocellulose membrane and presence of α-LA detected. Yellow dots obtained for bovine whey, commercial baby feed and milk powder signifie the presence of α-LA in these samples (Fig. 8a). ELISA was performed parallel to dot blot. Bovine whey showed highest content of α-LA, while the baby feed contained two times higher concentration of α-LA than the commercial milk powder (Fig. 8b).

## Discussion

Most applications of BsAbs require appreciable quantities of the proteins, while the currently available technologies including those based on recombinant DNA and hybridomas suffer from the limitation of poor yields^25^,^26^. The first report of production of BsAb involved chemical recombination of antibodies with two different specificities^8^. Despite the several improvements in the technology of joining of half antibody molecules/fragments via disulfide^12^ or other linkages^13^, presence of multiple functional groups in the antibodies interfere with the formation of target crosslinks^27^,^28^. Nonetheless, efforts to refine the chemical procedures to obtain BsAbs continue, including those in combination with recombinant procedures^29^. More recently a simple procedure for the production of BsAbs from a mixture of monoclonal antibodies by reduction of inter-heavy chain disulfides followed by their reoxidation was described^16^. The procedure subsequently employed to generate BsAbs both from monoclonal and polyclonal antibodies^5^ however gives poor yields.

Studies employing redox procedures for the BsAb production use reducing agents to selectively cleave inter-heavy chain disulfides in antibody mixtures leading to preparations that migrate as half molecules in the SDS gels. The mixtures are subjected to reoxidation of the thiols to accomplish the formation of BsAbs. The migration of the reduced antibody as a 75 kDa peptide in SDS gels is erroneously taken as dissociation of the molecule into two halves, whereas the dissociation may only occur in the gels due to presence of the detergent. The poor yields of BsAbs achievable in the chemical procedures used for their preparation^25^ can be attributable in our opinion, to poor dissociation of reduced parent antibodies into half molecules and consequently to low hetero dimerization during reoxidation of the sulfhydryl groups. It is well recognized that the heavy chains of IgG interact strongly through non-covalent linkages in the Fc region involving primarily the CH3 domain. In fact, direct evidence that shows that IgG molecules with reduced inter- heavy chain disulfides continue to behave like bivalent intact IgG is available^20^. That the strong association between the CH3 domains of IgG restricts the exchange of half molecules, is also supported by the studies on IgG4 antibodies. The IgG4 occur naturally as BsAbs in circulation^30^ due to their ability to undergo Fab arm exchange *in vivo*^23^.

Rabbit and goat IgG have a single disulfide bond linking the two heavy chains^31^,^32^ (Supplementary Fig. S5). These disulfides are more prone to reduction than those that join heavy and light chains^18^. Hong & Nisonoff^18^ have shown that the stabilities of the two types of inter-chain disulfide bonds joining heavy-heavy and heavy-light chains in rabbit IgG differ such that conditions of reduction can be defined for the preferential reduction of the inter heavy chain disulfide bond with minimal cleavage of disulfide bonds joining heavy and light chains. Pilot studies were therefore conducted using rabbit IgG. Selective reduction of the IgG (Supplementary Fig. S6) giving rise to a species that migrated primarily as ~75 kDa peptide in SDS-PAGE under non-reducing conditions was possible using either β-ME (Fig. 1a) or sodium sulphite (Fig. 1c). The preparation with reduced inter-heavy chain disulfides and blocked free sulfhydryls however coeluted with IgG in a Sephacryl S-200 gel filtration column suggesting a molecular mass indistinguishable from that of intact IgG (150 kDa) (Fig. 2a). It however migrated as a 75 kDa polypeptide in SDS-PAGE (Fig. 2a inset). This suggests that the IgG with reduced inter-heavy chain disulfides dissociated into half molecules only in presence of SDS (Supplementary Fig. S6). This was substantiated by performing the gel filtration in presence of SDS (Fig. 2b). SDS causes partial unfolding and markedly increases the hydrodynamic radius of proteins, for this reason it was necessary to use Sephacryl S-300, a gel filtration matrix with very high fractionation range (fractionation range of 1500 kDa)^33^. IgG with reduced inter chain disulfides and blocked free sulfhydryls emerged from the Sephacryl S-300 column with an elution volume that corresponded to a molecular mass of 79 kDa, while IgG emerged earlier (Fig. 2b).

Considering the risk of irreversible protein denaturation in presence of SDS^34^, resilience of rabbit IgG against exposure to SDS was investigated. As shown in Fig. 4 exposure to SDS up to a concentration of 0.1% caused only a moderate loss in secondary/tertiary structure, while in presence of 0.25% SDS, marked denaturation was evident. At very low concentration of surfactants, electrostatic interactions prevail between protein and surfactant, whereas at higher concentration, hydrophobic interactions tend to dominate. These interactions solely depend on the chain length of hydrophobic region in the surfactant. SDS has considerably long hydrophobic tail, facilitating its access to the hydrophobic domains of proteins^35^. Apparently, at concentrations above 0.1%, SDS disrupts the hydrophobic interactions and causes the unfolding of IgG, as revealed by the UV CD and intrinsic fluorescence spectra (Fig. 4c–e). Similarly, the ability of the anti-α-LA IgG and anti-HRP IgG to bind to the respective antigens was only moderately lowered by exposure to 0.1% SDS, but decreased remarkably when treated with 0.25% SDS. A previous study also showed that IgG lose their antigen precipitating activity in presence of 0.2% SDS, while the activity is retained at lower concentrations of the detergent^36^. It is possible that exposure to 0.2% or higher concentration of SDS irreversibly denatures the antigen binding sites by inducing a major conformational change.

Having established that SDS can be used to dissociate the antibodies with reduced inter-heavy chain disulfide into two half molecules and the concentration of the detergent does not cause significant loss in antigen binding ability, we proceeded with the preparation of BsAbs from anti-α-LA and anti-HRP antibodies using the modified redox procedure in which reaction mixture additionally contained 0.1% SDS. It was envisaged that SDS, by facilitating the separation of reduced antibodies into half molecules will enhance their ability to interact with other half molecules and form BsAbs. Our results showed that inclusion of SDS in the mixture of reduced antibodies followed by removal through dialysis indeed increased the yield of BsAbs formation dramatically (Fig. 5). It was also possible to successfully use IEC fractions of IgG isolated from the sera of rabbits immunized against HRP and α-LA which are likely to contain large fraction of non-specific IgG in addition to those recognizing the antigens used for immunization in the experiments. This would greatly lower the probability of formation of BsAbs containing one arm recognizing HRP and other α-LA. Formation of significant quantities of the BsAbs under the conditions however shows that SDS, by dissociating the mixtures of reduced antibodies to half molecules, greatly facilitates their re-association to form a heterogeneous mixture of IgG including the BsAbs recognizing HRP and α-LA. Significantly, inclusion of SDS also caused marked increase in the BsAb formation when the partially purified goat anti-HRP and anti-HSA antibodies were used for the preparation. The modified redox procedure was also successful in the preparation of hybrid bifunctionals from rabbit anti-HRP and goat anti-HSA IEC fraction (Fig. 6).

Immuno affinity purification of antibodies is a harsh and low-yield procedure that can also cause significant loss of antibody activity. Antigen antibody interactions are usually very strong necessitating exposure to low pH for their dissociation that may cause conformational alterations as well as degradation and aggregation of certain antibodies^37^. Use of mild elution condition and/or low affinity matrices have been proposed to alleviate the damage^38^. Since synthesis of BsAbs using chemical procedures requires highly purified preparations (usually using affinity chromatography) of the two antibodies and twin affinity purification steps to isolate the pure BsAbs, final yields are generally quite poor. To address the issue, Paul *et al*.^39^ prepared BsAbs by subjecting the culture supernatants of Human IgG 1 and Kappa antibodies (with point mutations) to Fab arm exchange in the unfractionated culture supernatant followed by affinity purification of the resulting bifunctional.

Purification of the BsAb prepared from 100 mg of rabbit anti-α-LA and anti-HRP antibodies from the dual affinity chromatography yielded 0.5 mg of protein with enrichment of about 200-fold in the antigen binding activity (Fig. 6). The purified preparation bound fully to affinity supports bearing covalently coupled α-LA or HRP, suggesting purity of the bifunctional. The modified redox procedure is rapid, results in good yields and it takes only 2–3 days for obtaining the BsAbs from IEC fraction. Also the procedure is quite amenable to scaling up in view of the small number of steps involved and offers the possibility of preparing BsAbs even in a resource constrained laboratory (Fig. 7). Procedures employing redox methods or genetic engineering for generating IgG format BsAbs suffer from low yields. Several attempts have also been made earlier to weaken the interaction between the Fc regions of the antibody molecules to facilitate their dissociation and formation of BsAbs which includes knob into hole strategy^40^, common light chain approach^41^, combining CrossMab with knob into hole strategy^42^, half molecule exchange^43^, κλ bodies^44^ and preferential species-restricted heavy chain pairing^45^. The methodology described here offers a simpler and inexpensive alternative.

The anti-α-LA: anti-HRP BsAb could be used successfully in the assay of α-LA in biological samples, both by ELISA and dot blot (Fig. 8). Immunization with α-LA has been proposed as strategy for prevention of development of breast cancer^46^ and the BsAbs may be useful in the monitoring of the level of protein during the immunization and possible indicator of breast tumors^47^. A multitude of other BsAb formats with one arm recognising an enzyme such as HRP, alkaline phosphatase or galactosidase have been used for detection of cancer markers and bacterial antigens as reviewed by Cao *et al*.^48^.

## Conclusion

The preparation of BsAbs for clinical application by the existing procedures has been stymied by the challenges faced in the production such as high production cost, requirement of sophisticated laboratory set up and low production yields. A modified redox procedure that remarkably improves the yield of BsAb over the conventional procedure is described in this study. By employing a detergent to facilitate dissociation of reduced antibody into half molecules, BsAbs can be prepared in high yields. The procedure also offers the possibility of preparing BsAbs from partially purified antibodies, also derived from different species. Using this procedure BsAbs can be produced in bulk rapidly even in a resource constrained laboratory.

## Methods

### Ethics statement

All experiments were approved by the Aligarh Muslim University Bioethical Committee. Healthy rabbits were used for immunization and blood was collected from the animals maintained at the animal house of the Interdisciplinary Biotechnology Unit, Aligarh Muslim University, in accordance with the recommendations of Committee for the Purpose of Control and Supervision of Experiments on Animals (Registration No 332, CPCSEA), Ministry of Environment and Forest, Government of India.

### Chemicals and reagents

All the chemicals and reagents used were of the highest purity available. α-LA, HRP, β-ME, DEAE-cellulose, Sepharose-4B, Sephacryl S-300, Sephacryl S-200, Freund’s complete and incomplete adjuvants, bicinchoninic acid (BCA) protein estimation kit and Tween-20 were purchased from Sigma-Aldrich Chemicals (St Louis, MO, USA) and used as received. Anti-HSA and anti-HRP goat IgG were purchased from MP Biomedicals, Mumbai, India. DTNB was purchased from Himedia, Mumbai, India. HRP-conjugated goat anti-rabbit IgG was purchased from Bangalore Genei Pvt. Ltd. (Bangalore, India), Protein concentrators (Amicon Ultra-15 centrifugal filter units) were purchased from Merck Millipore, India. Milk powder (Nestle Everyday, India), Baby feed (Nutricia Dexolac, India.) and raw bovine milk were purchased from the local grocery store. Whey was isolated from raw bovine milk by acid precipitation of casein. SDS and other reagents used were of analytical grade.

### Animal immunization and antibody purification

Eight to ten weeks old rabbits were used as hosts for raising antibodies. Primary immunization was executed by injecting them with 150 μg of the antigens (α-LA or HRP) emulsified with complete Freund’s adjuvant. The first booster contained 100 μg of the antigens as incomplete Freunds adjuvant emulsion and administered after 21 days of first immunization. On the fifth day of booster, the animals were bled, sera collected and decomplemented by a brief exposure to 50 °C. Gamma globulin were precipitated by 40% ammonium sulphate saturation^49^ and purified by negative IEC using DEAE-cellulose^50^. The purification was assessed using 10% SDS-PAGE gels both under reducing and non-reducing conditions^51^.

### Preparation of affinity columns

α-LA and HRP were separately coupled to cyanogen bromide (CNBr)-activated Sepharose 4B for the preparation of the affinity supports^52^. Briefly, 10 g Sepharose 4B was washed with distilled water followed by the addition of 2 M Na_2_CO_3_ solution. Further, 1.0 g CNBr dissolved in 1.0 ml acetonitrile was added with constant stirring at 4 °C. After 15 min, the activated gel was washed with the coupling buffer (0.1 M bicarbonate buffer, pH 8.5), followed by distilled water and again with the coupling buffer. The washed gel was suspended in the coupling buffer containing the respective proteins to facilitate their binding by slow stirring at 4 °C. Unbound protein in the supernatant and washes was quantitated. Remaining active groups on the Sepharose matrix were blocked by incubating with 1 M Tris pH 7.4 for 2 h at room temperature (RT).

Specific antibodies were purified using the respective affinity matrices packed in columns (1 cm × 5 cm). The affinity matrices were washed with 3 bed volumes (BV) of 2.0 M arginine-HCl, pH 2.7, subsequently neutralized by washing with PBS (20 mM sodium phosphate buffer, 154 mM sodium chloride, pH 7.4) and 3 BVs of 0.1 M sodium phosphate, pH 9.0 and again neutralized with PBS, pH 7.4. The IEC fractions were concentrated and then allowed to bind to the affinity matrices by circulating them 3 times through the columns. The unbound proteins remaining in the column were removed by washing with PBS, pH 7.4 until the wash fraction reached 0.05 OD at 280 nm. The matrix-bound antibodies were eluted using 2 M arginine-HCl, pH 2.7 and the pH of elute was neutralized immediately by the addition of a 1/4 volume of 1.5 M Tris^53^. The eluted fractions were dialyzed against 3 changes of PBS, pH 7.4 at 4 °C and concentrated to 2 mg/ml using a concentrator (Merck Millipore) with cut off range 10 kDa.

### Optimizing conditions for generating antibody half molecules

Antibody half molecules were generated by two different procedures. Two mg/ml rabbit antibody solutions were reduced with varying concentration of [i] sodium sulphite (200–500 mM) in presence of 2.5 mM DTNB in Tris EDTA (TE) buffer (20 mM Tris, 1 mM EDTA, pH 8.8) for 2 h at 37 °C followed by dialysis against the same buffer and [ii] β-ME (0–200 mM) in Tris buffer for 1 h at 37 °C. The formation of half molecules was assessed by non-reducing SDS-PAGE. Similarly, goat IgG were reduced with β-ME to obtain half molecules.

### Gel filtration chromatography

A 150 ml glass column with an internal diameter of 1.8 cm was packed with 140 ml Sephacryl S-200 resin. The column was washed with 3 BVs of Tris buffered saline with a flow rate maintained at 15 ml/h. The column was then calibrated by passing 5–6 mg of different protein markers. Five milligrams of DTNB treated half molecules were then loaded onto the column.

To study the effect of SDS on separation of half molecules, a 100-cm-long glass column with an internal diameter of 2 cm, was packed with about 240 ml preswollen Sephacryl S-300, and equilibrated with Tris buffered saline containing 0.1% (w/v) SDS. The flow rate was adjusted to 15 ml/h and 1.5 ml fractions were collected. The column was calibrated with protein markers. Absorbance of the collected fractions was recorded at 280 nm using Perkin Elmer UV/VIS spectrometer model lambda 25 and plotted against the elution volume.

### Dynamic Light Scattering (DLS) measurement

Ten μM solutions of native IgG or half molecules in presence of 0.1% (w/v) SDS in Tris EDTA buffer, pH 8.8 were subjected to DLS measurement to determine the hydrodynamic radii (*R*_*h*_) and corresponding molecular weights. The *R*_*h*_ was measured using DynaPro-TC-04 dynamic light scattering instrument (Protein Solutions, Wyatt Technology, Santa Barbara, CA) equipped with temperature controlled microsampler. Samples were directly filtered through 0.22 μm pore sized filters in quartz cuvette and *R*_*h*_obtained by recording dynamic light scattering at 830 nm. The *R*_*h*_ shown was an average of 20 measurements. Mean *R*_*h*_ and polydispersity were estimated by auto correlation analysis of the scattered light intensity, based on translational diffusion coefficient, from the Stokes-Einstein equation^54^.


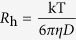


where *R*_*h*_ is the hydrodynamic radii (nm), k is the Boltzmann’s constant, T is the absolute temperature (K), η is the viscosity of water and D is the translational diffusion coefficient^54^.

### Effect of SDS on antigen binding activity of antibodies

Retention of antigen binding activity by anti-α-LA IEC fraction on exposure to SDS was investigated. Two milligrams of anti-α-LA antibodies (IEC fraction) were incubated for 1 h at 37 °C with varying concentrations of SDS [0%, 0.03%, 0.05%, 0.1% and 0.25% (w/v)] in a total volume of 1.0 ml in TE buffer and dialysed against PBS, pH 7.4 for 24 h at 30 °C. Similarly IEC fraction of rabbit anti-HRP and goat anti-HRP antibodies were also examined for effect of SDS.

### Near and Far UV CD Analysis

CD measurements were carried out using Jasco spectropolarimeter (J-815) equipped with thermostatically controlled cell-holder attached to a Peltier with Multitech water circulator. The instrument was calibrated with D-10 camphor sulfonic acid. Protein concentrations taken were 13.2 μM for near UV CD and 3.3 μM for far UV CD measurements of each test sample and spectra were recorded using a cell of 0.1 cm path length with scan speed of 100 nm/min and response time of 2 s. Each spectrum shown is the average of two scans. All spectra were smoothed by the Savitzky-Golay method with 25 convolution width.

### Intrinsic Fluorescence Analysis

To study the effect of exposure to SDS on tertiary structure of IgG, tryptophan fluorescence spectra were recorded on a Shimadzu RF-5301 PC spectro fluorophotometer. The spectra were measured at 25 ± 0.1 °C with a 1 cm path length cell. Protein concentration taken was 3.3 μM, and fluorescence was measured by exciting the protein at 295 nm, and emission spectra were recorded in the range of 300–500 nm. Both the excitation and emission slit were set at 5 nm.

### ELISA for monospecific and bispecific antibodies

Ninety six welled microtitre plates were coated overnight at 4 °C by adding 10 μg of α-LA in 100 μl of coating buffer (0.1 M bicarbonate buffer, pH 9.5) in each well. The α-LA coated plates were washed with PBST (PBS, pH 7.4 with 0.05% (v/v) tween-20) and blocked by adding 200 μl per well, PBS containing 5% (w/v) bovine serum albumin/2% (w/v) fat-free casein at 37 °C for 5 h or at 4 °C overnight. The plates were washed and subsequently aliquots of mono specific or BsAbs were added to the wells. The samples were two-fold serially diluted in PBS in subsequent wells and the plates further incubated at 37 °C for 2 h. This was followed by washing three times with PBST. The wells were further incubated with 100 μl of 10 μg/ml HRP in case of bispecific (Supplementary Fig. S7), and HRP conjugated goat anti rabbit secondary antibody in case of mono specific and the plates incubated at 37 °C for 1 h, washed again with PBST before the addition of 100 μl of substrate solution (10 mg OPD salt dissolved in 12 ml of 0.1 M citrate buffer with 10 μl of 30% H_2_O_2_) and were finally incubated at 37 °C for 1 h in case of bispecific, and for 15 min when mono specific were assayed. The reaction was terminated by the addition of 50 μl of 1.0 M H_2_SO_4_. Absorbance was recorded at 490 nm with a microtitre plate reader (Bio-Rad, Hercules, CA 94547 USA). Samples were tested in triplicate and results were expressed as mean of three independent experiments after subtracting the values of controls (Supplementary Table S2).

### Construction of BsAbs

Two mg/ml rabbit anti-α-LA and anti-HRP IEC fractions were reduced separately with 10 mM β-ME at 37 °C for 1 h, mixed in 1:1 molar ratio with or without added 0.1% SDS (w/v). Excess reducing agent and SDS were then removed by extensive dialysis against PBS at 30 °C. The BsAb content was determined by ELISA as described above (Supplementary Fig. S7). Similarly, goat anti-HSA: goat anti-HRP as well as goat anti-HSA: rabbit anti-HRP BsAbs were also prepared and their formation followed by ELISA.

### Purification of BsAbs from IEC fraction

Fifty milligrams each of rabbit anti-α-LA and anti-HRP IEC fractions were subjected to thiol reduction and oxidation in presence of SDS to obtain a crude solution containing BsAbs as described above. The resulting preparation containing the BsAbs was purified over using a α-LA-Sepharose column equilibrated with PBS. BsAbs along with monospecific anti-α-LA remained bound while, monospecific anti-HRP antibodies were collected in the wash fraction (Fig. 7). The column was eluted with 2 M arginine-HCl, pH 2.7, the pH of eluted fractions was immediately neutralized by the addition of a 1/4 volume of 1.5 M Tris and pooled. The pooled fractions were then dialyzed against PBS, concentrated and subjected to additional chromatography on a HRP-Sepharose column (1 cm × 5 cm) equilibrated with PBS, pH 7.4. Only the BsAbs were bound to the matrix which were eluted from the column, as monospecific anti-HRP antibodies were already removed when BsAbs were initially passed through the α-LA-Sepharose. Protein content in eluted fractions was monitored by absorbance at 280 nm at each step. Fractions were then pooled, dialyzed against PBS, pH 7.4 and concentrated. Bispecificity was tested at each step by ELISA.

### Dot blot assay

For immunodetection of antigen dots, appropriate quantities of the protein antigens dissolved in coating buffer were applied on the nitrocellulose strips with the help of a microsyringe. The strips were dried and blocked with blocking buffer for 2 h at RT, washed thoroughly with PBST, then the strips were incubated with BsAbs in PBS, pH 7.4 for 2 h at RT with continuous shaking. The strips were washed again and HRP was used for detection as described for ELISA. After washing, OPD substrate solution was added and colour developed in dark. The strips were then thoroughly washed with distilled water and allowed to dry at RT.

### Multiplicity of the experiments

All experiments were carried out multiple times and values shown are mean of at least three independent closely agreeing experiments.

## Additional Information

**How to cite this article**: Gupta, J. *et al*. A detergent-based procedure for the preparation of IgG-like bispecific antibodies in high yield. *Sci. Rep.* **6**, 39198; doi: 10.1038/srep39198 (2016).

**Publisher's note:** Springer Nature remains neutral with regard to jurisdictional claims in published maps and institutional affiliations.

## Supplementary Material

Supplementary Information
