# Supplementary material for: A detergent-based procedure for the preparation of IgG-like bispecific antibodies in high yield
Source: Sci Rep. 2016 Dec 16;6:39198. doi: 10.1038/srep39198 (PMC5159798; doi:10.1038/srep39198)
Supplement: Supplementary Information [file srep39198-s1.pdf]

A detergent-based procedure for the preparation of IgG-like bispecific antibodies in high yield

Jyoti Gupta, Mehboob Hoque, Masihuz Zaman, Rizwan Hasan Khan, M. Saleemuddin*

Interdisciplinary Biotechnology Unit

Aligarh Muslim University,

Aligarh 202002, India.

*Corresponding author, Tel.: +91 9897179733; Fax: +915712721776

Email address: msaleemuddin47@gmail.com

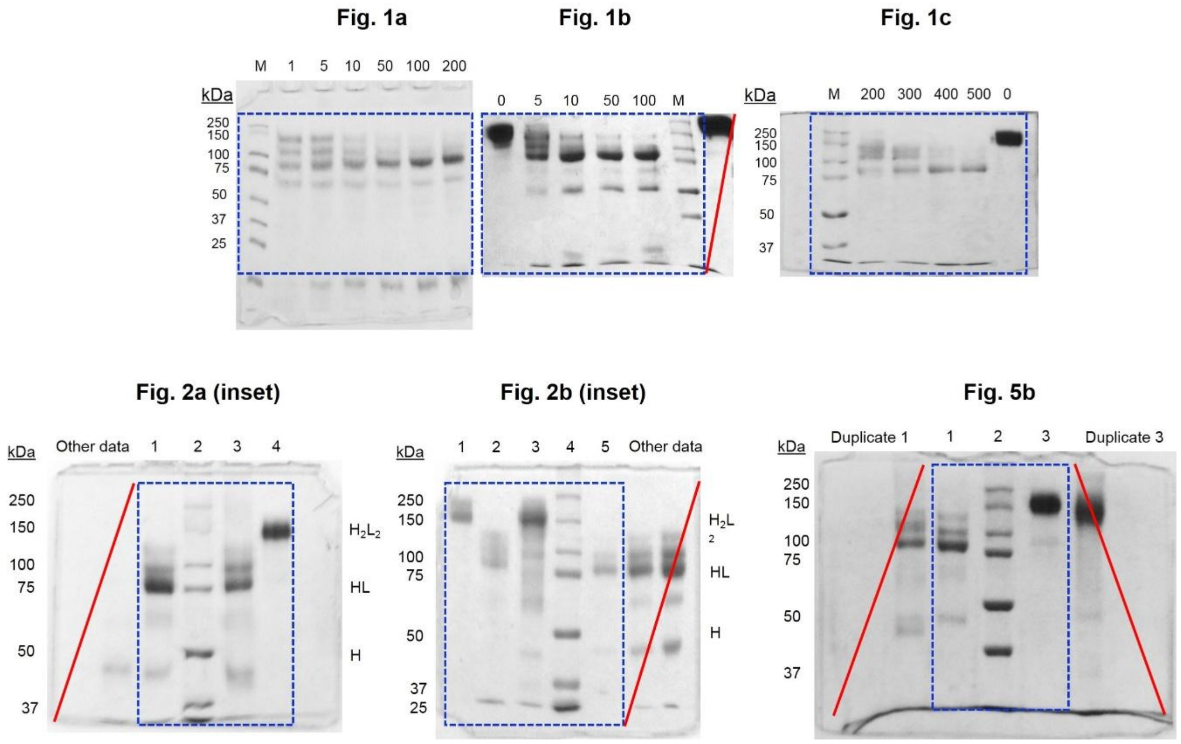

Supplementary Figure S1. Representative full size gel images of SDS-PAGE. Full length original images of the gels presented in the corresponding main figures. The blue dotted boxes indicate cropped regions.

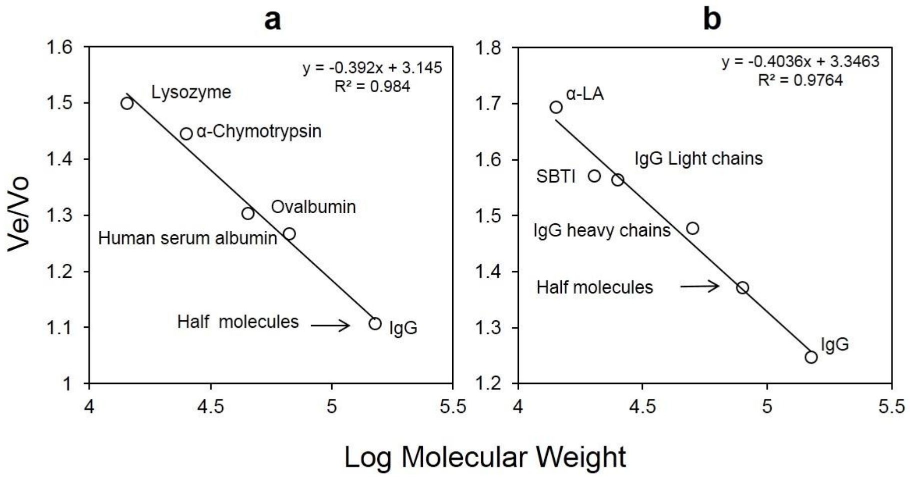

Supplementary Figure S2. Calibration curve for gel filtration columns; Sephacryl S-200 in absence of SDS (*Panel* ***a***) and for Sephacryl S-300 in presence of SDS (*Panel* ***b***).

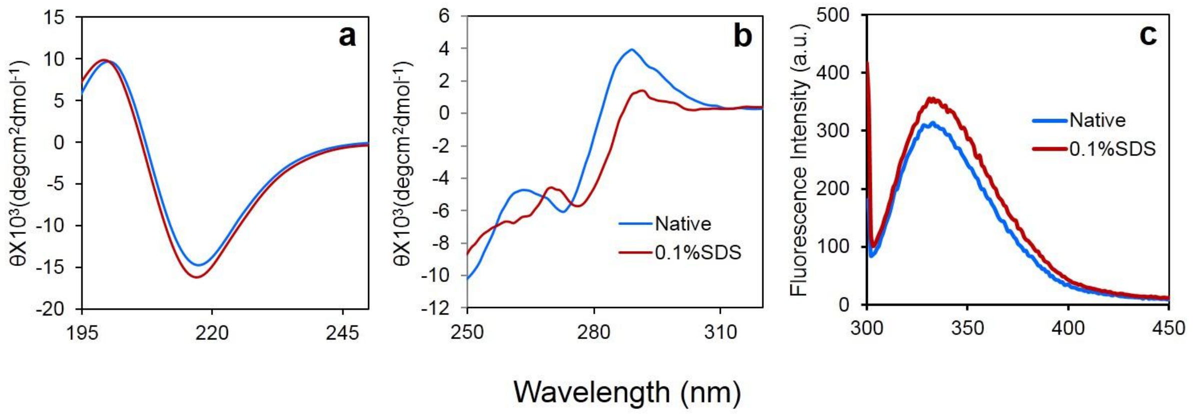

Supplementary Figure S3. UV CD and intrinsic fluorescence spectra of goat IgG exposed to 0.1% (w/v) SDS. Goat IgG were incubated in presence and absence of 0.1% (w/v) SDS for 1 h, dialysed extensively against PBS, pH7.4 and subjected to far-UV CD (*Panel **a***), near-UV CD (*Panel **b***) and intrinsic fluorescence (*Panel **c***) spectral analysis.

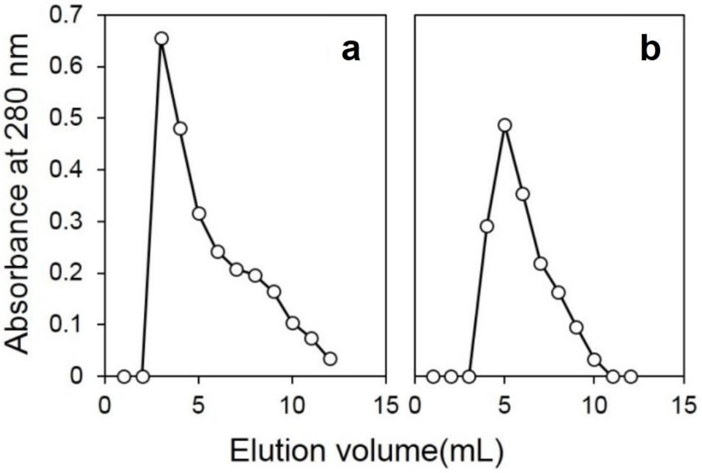

Supplementary Figure S4. Elution profiles of BsAb samples prepared from IEC fractions of rabbit anti-HRP and rabbit anti-α LA antisera. The sample containing BsAbs was passed through a Sepharose-α-LA column (*Panel* ***a***) and the active fractions pooled, dialyzed and further purified on a Sepharose-HRP column (*Panel* ***b***).

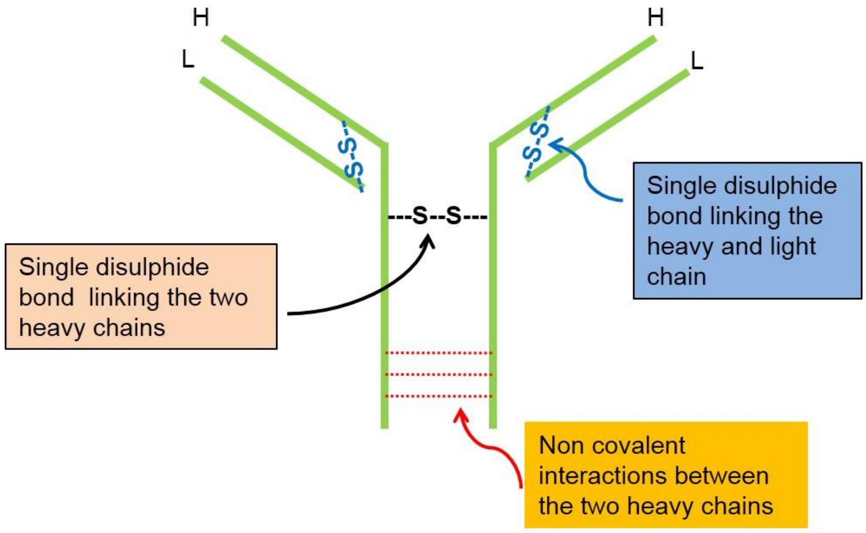

Supplementary Figure S5. Interchain disulphide bond structure for rabbit/goat IgG.

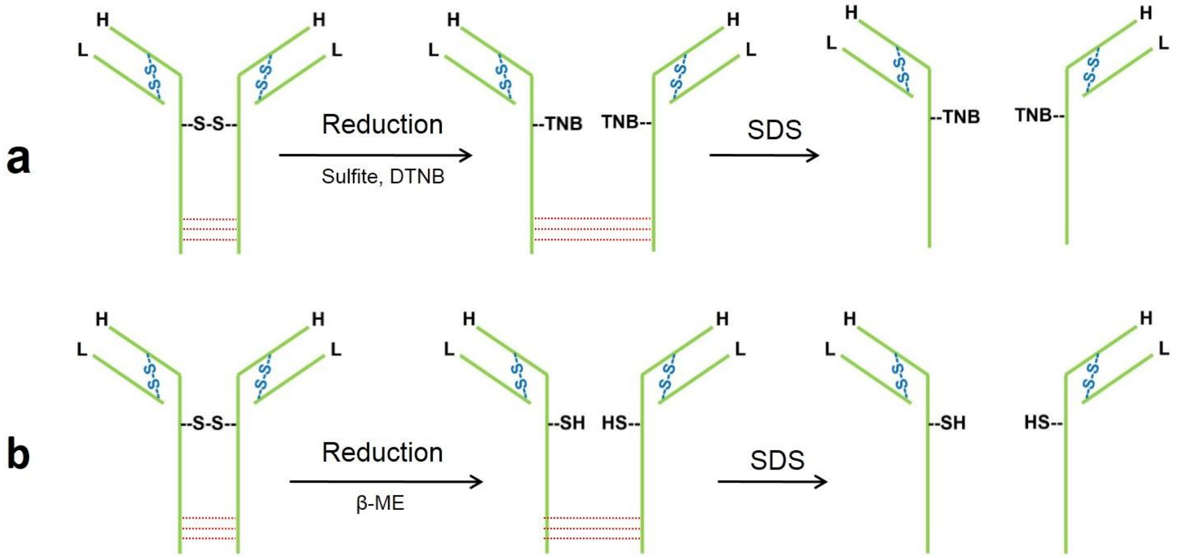

Supplementary Figure S6. Schematic presentation of reduction of rabbit IgG into half molecules using different thiol reductants. Selective reduction of inter heavy chain disulphide bond of rabbit IgG and subsequent modification of thiol groups to thio-nitrobenzoate (TNB) groups, followed by separation into half molecules in presence of SDS (*Panel* ***a***). Selective reduction of inter heavy chain disulphide bond of rabbit IgG to free thiol (SH) group followed by separation into half molecules in presence of SDS (*Panel* ***b***).

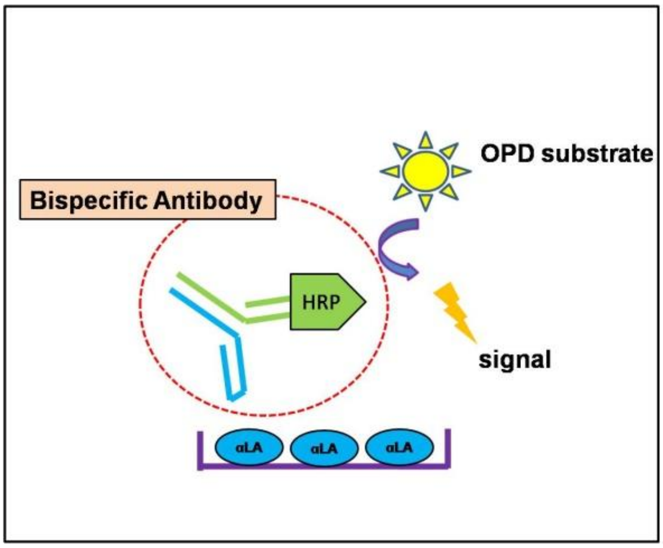

Supplementary Figure S7. Schematic presentation of assay of anti-α-LA: anti-HRP BsAbs by ELISA.

	Hydrodynamic Radii (nm)	Percent Polydispersity	Mol. Wt. (kDa)
**Native IgG**	5.4 ± 0.3	10.1	151
**IgG Half molecule**	3.4 ± 0.2	10.9	79

Supplementary Table S1. Different parameters obtained from DLS measurements.

Control	α-LA Coating	Anti-α-LA antibodies	Anti-HRP antibodies	Anti-α-LA: Anti-HRP BsAbs	HRP	OPD substrate solution
**1.**	-	-	-	+	+	+
**2.**	+	-	-	-	-	+
**3.**	+	+	-	-	+	+
**4.**	+	-	+	-	+	+
**5.**	+	-	-	-	+	+
**6.**	+	-	-	+	-	+

Supplementary Table S2. Tabular presentation of controls used in assay of BsAb by ELISA. The wells were blocked with BSA after coating with α-LA (in control 1without addition of α-LA) and they were washed thoroughly between various steps.
